# Integration of a miniaturized DMMB assay with high-throughput screening for identifying regulators of proteoglycan metabolism

**DOI:** 10.1038/s41598-022-04805-y

**Published:** 2022-01-20

**Authors:** Yi Sun, Yuen-kee Tsui, Mengqi Yu, Minmin Lyu, Kenneth Cheung, Richard Kao, Victor Leung

**Affiliations:** 1 Department of Orthopaedics and Traumatology, The University of Hong Kong, Hong Kong, China; 2 Department of Diagnostic Radiology, National University of Singapore, Singapore, Singapore; 3 Core Laboratory, The University of Hong Kong Shenzhen Hospital, Shenzhen, China; 4 Department of Microbiology, The University of Hong Kong, Hong Kong, China

**Keywords:** High-throughput screening, Cartilage, Drug development, Assay systems

## Abstract

Defective biosynthesis or function of proteoglycans causes pathological conditions in a variety of tissue systems. Osteoarthritis (OA) is a prevalent degenerative joint disorder characterized by progressive cartilage destruction caused by imbalanced proteoglycan synthesis and degradation. Identifying agents that regulate proteoglycan metabolism may benefit the development of OA-modifying therapeutics. High-throughput screening (HTS) of chemical libraries has paved the way for achieving this goal. However, the implementation and adaptation of HTS assays based on proteoglycan measurement remain underexploited. Using primary porcine chondrocytes as a model, we report a miniaturized dimethyl-methylene blue (DMMB) assay, which is commonly used to quantitatively evaluate sulfated glycosaminoglycan (GAG) content, with an optimized detection range and reproducibility and its integration with HTS. Treatment with TGF-β1 and IL1-α, known as positive and negative proteoglycan regulators, respectively, supported the assay specificity. A pre-test of chemical screening of 960 compounds identified both stimulators (4.48%) and inhibitors (6.04%) of GAG production. Fluorophore-assisted carbohydrate electrophoresis validated the activity of selected hits on chondroitin sulfate expression in an alginate culture system. Our findings support the implementation of this simple colorimetric assay in HTS to discover modifiers of OA or other diseases related to dysregulated proteoglycan metabolism.

## Introduction

Proteoglycans are macromolecules containing a core protein backbone to which one or more sulfated glycosaminoglycans (GAGs) are covalently attached, mostly in the form of chondroitin sulfate (CS), keratin sulfate and heparin sulfate chains. Proteoglycans are capable of sequestering water molecules and establishing swelling pressure via hydrophilic GAG, thereby providing mechanical strength against compressive forces. In addition, proteoglycans play pivotal developmental and homeostatic functions in both extracellular matrix organization^1^ and cell behaviour regulation^2–4^ by deploying various growth factors (e.g., TGF-β^5^ and FGF^6^), cytokines (e.g., interleukin-10^7^ and CCL5^8^), and other distinct signalling molecules (e.g., hedgehogs^9^ and STING^10^) via their chemical binding to GAGs.

Degenerative joint diseases, such as osteoarthritis (OA) and intervertebral disc degeneration, are leading causes of disability and present significant challenges in clinical management^11,12^. Typical treatments are mostly palliative, underscoring the need to develop disease-modifying drugs to mitigate the conditions. Notwithstanding its multifactorial nature^12,13^, the structure and functional failure of the joints is tightly associated with the loss of proteoglycans in the cartilaginous elements^14,15^. Proteoglycans, in particular the large chondroitin sulfate-rich proteoglycan aggrecan, contribute to structural and mechanical integrity^15^. Aggrecan degradation by ADMTS-5 is one of the key features of OA pathogenesis^15^, and the resulting 32-mer fragment could drive osteoarthritic pain^16^. The involvement of small leucine-rich proteoglycans (SLRPs)^17^, such as matriline^18^, biglycan^19^ and fibromodulin^20^, has also been reported. As such, strategies for probing regulators of proteoglycan metabolism in chondrocytes could be valuable to the understanding and treatment of OA.

Phenotypic screening opens up a new research avenue for identifying agents of pharmacological potential. Previous studies explored regulators of chondrogenesis in mesenchymal stromal cells^21^ and ATDC5 cells^22^ by assessing proteoglycan content by Alcian blue staining in high-throughput screening (HTS). However, imaging-based assays are semiquantitative and therefore are subject to high false discovery rates. Because of its simplicity and sensitivity, the dimethylmethylene blue (DMMB) assay has been commonly used for GAG measurement to determine proteoglycan content^23^. Using porcine chondrocytes as a model, we report an optimized, miniaturized DMMB assay-based GAG detection system and its integration with a robotic HTS platform to enable chemical library screening of potential proteoglycan metabolism modifiers.

## Results

### Miniaturization of the DMMB assay

**Figure 1:**
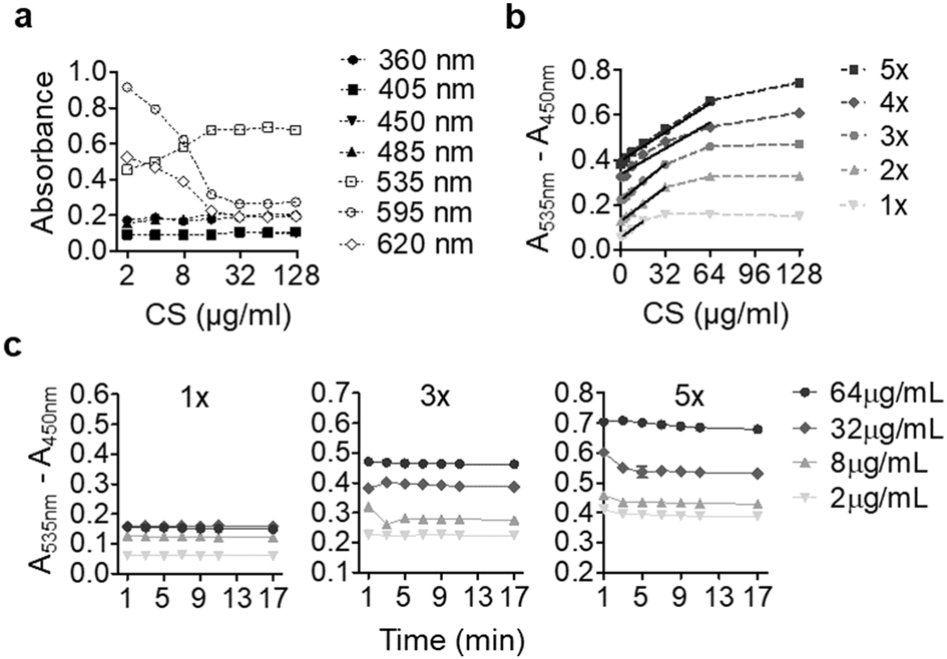
Optimizing GAG detection in a miniaturized DMMB assay. (**a**) Absorbance profile of the DMMB reaction with a range of chondroitin sulfate concentrations (CS, 2–1128 μg/mL). (**b**) Effects of different concentrations of DMMB reagent (1× refers to typical concentration) on the linear reading window. (**c**) Kinetics of the DMMB assay at specific concentrations of chondroitin sulfate using different concentrations of DMMB reagent. Data are expressed as the mean ± SEM from five independent experiments.

Primary HTS generally employs an assay system with a sufficient detection range and sensitivity to maximize the probability of hit identification. We set out to investigate DMMB assay conditions in a miniaturized, 96-well format that enables maximal detection of CS, the major GAGs expressed in chondrocytes. At wavelength A450, measurement of arbitrary units of CS was not affected by either the reaction or autofluorescence from the culture plate (Fig. 1a), providing an appropriate reference wavelength. Consistent with previous findings^24^, absorbance at 535 nm was positively related to the DMMB-CS complex but was inversely correlated to DMMB consumption, as indicated at 595 nm (Fig. 1a). We tested different concentrations of DMMB dye in the reaction and found that a concentration fivefold higher than the conventional method could substantially extend the linear detection window (Fig. 1b). This corresponds to an increase in the upper detection limit from 16 to 64 μg/mL without affecting sensitivity (R^2^ = 0.993) (Supplementary Table S1). Assay kinetics showed relatively stable readouts between 5 and 16 min after the start of the reaction (Fig. 1c).

### Adaptation of chondrocyte culture in a miniaturized assay

**Figure 2:**
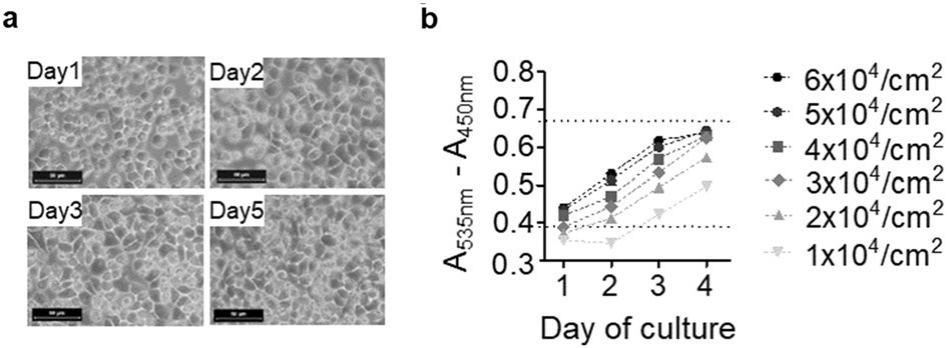
Performance of miniaturized DMMB assays with primary chondrocyte culture. (**a**) Primary chondrocytes from porcine costal cartilage showed polygonal morphology and reached full confluence at Day 5. (**b**) DMMB assay with chondrocytes at different seeding densities (1–6 × 10^4^/cm^2^) and culture periods. Dotted lines indicate the linear reading range (0.37–0.69). Values are presented as the mean ± SEM. DMMB (n = 5); scale bar, 50 µm.

Cell-based screening requires optimized culture conditions. Phenol red, as a typical pH indicator in culture medium, was removed to minimize the influence on the absorbance attributed to the colour change due to pH change. To minimize cell dedifferentiation under monolayer culture and maximize proteoglycan accumulation without the need to refresh the culture medium, primary porcine chondrocytes were cultured in 384-well plates at a high density for 3 days (Fig. 2a). A seeding density of 3 × 10^4^ cells/cm^2^ achieved 90% confluency by Day 3, with a round or polygonal cell morphology. To facilitate HTS applications, DMMB reagent (5×) was directly added to the culture wells (instead of collecting the medium) for reaction. Measurements yielded readouts in the mid-range of the calibration curve (A_535nm_ − A_450nm_ = 0.533, Fig. 2b). Additionally, an MTT assay was used to assess changes in overall cellular activity. The measurement (A_570nm_ − A_630nm_) showed a linear increasing trend with longer incubation time and reached a plateau stage after 24 h (Supplementary Fig. S1).

### Response to TGF-β and IL1-α induction

**Figure 3:**
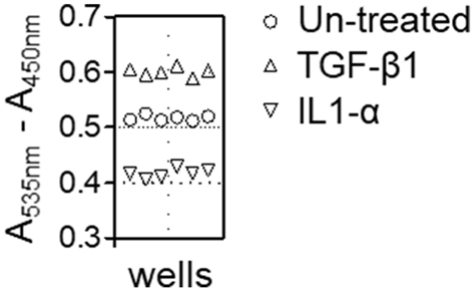
Detection of cytokine-mediated activity. Proteoglycan production in primary chondrocytes treated with TGF-β1 (10 ng/mL) or IL1-α (10 ng/mL) for 3 days was evaluated by the miniaturized DMMB assay. Readouts were recorded as the average of six reactions (wells) from three independent plates.

With the protocol established (Supplementary Fig. S2), we further evaluated the robustness of the system in detecting the stimulation and inhibition of proteoglycan production by TGF-β^25^ and IL1-α^26^, respectively. The results showed distinct readout separation in the upper and lower linear ranges (Fig. 3). Analysis of strictly standardized mean differences indicated a Z-factor of 0.52 (TGF-β) or 0.53 (IL1-α), comparable to the value expected in “excellent” HTS-ready assays^27^.

### Assay validation

**Figure 4:**
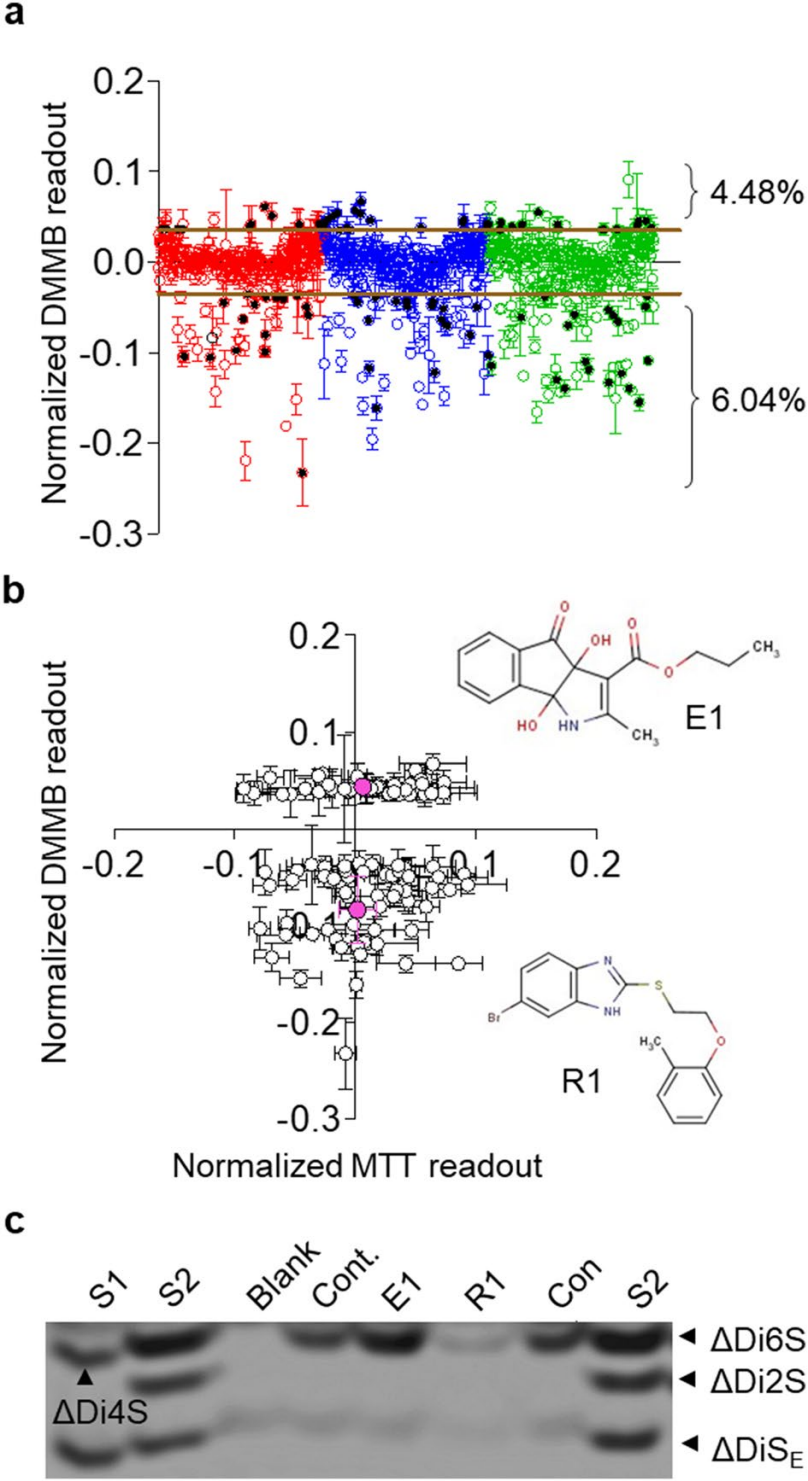
Performance of 960 compounds in the HTS DMMB assay. (**a**) Scatter plot of DMMB assay readouts from 960 compounds. The median of each plate was used for subtraction normalization. The primary screen activities were identified with readings greater or lower than the plate median + 3 SD. Each batch contained 320 compounds and was performed in triplicate. Hits were highlighted by black dots. (**b**) Array of hits compounds in MTT assay. Insert: chemical structures of an enhancer (E1, upper) and a repressor (R1, lower) compound. (**c**) E1 and R1 were tested in primary chondrocytes cultured in a 3D alginate culture system. Analysis of compositional disaccharides by fluorophore-assisted carbohydrate electrophoresis (20% acrylamide) indicated regulation of chondroitin sulfate GAG expression. Standards are shown in the margins (arrow). S1: ΔDi4S, ΔDiS_E_; S2: ΔDi6S, ΔDi2S, ΔDiS_E_.

We conducted a pilot study to test the effectiveness of the optimized, miniaturized assay system in screening a chemical library of 960 compounds in a robotic HTS platform (Fig. 4). Analysis of the mean for controls (0.1% DMSO) from 30 wells covering marginal and middle regions in each plate of a total of nine plates showed an overall CV (coefficient of variation) < 10%, supporting plate-to-plate comparison and that edge effect or drift of assay readings were negligible (Table 1). S–W values were mostly greater than 0.05, suggesting a normal distribution of readings. The Z′ score of the medium control within each plate varied from + 2.59 to − 2.03, indicating few deviations and steady assay readings (Supplementary Fig. S3). Midpoints of classes of datasets showed no drift.

**Table 1:** Statistics of culture controls in the chemical screening.

Statistics calculation based on	Mean	SD	% CV	S–W	Midpoint
Readings from the same well position of nine plates (15 wells from marginal area were deployed), n = 9	0.506	0.017	3.3	0.646	0.4985
	0.521	0.010	1.8	0.047	0.527
	0.519	0.014	2.7	0.679	0.5165
	0.535	0.012	2.2	0.059	0.539
	0.522	0.017	3.3	0.901	0.518
	0.514	0.010	1.9	0.464	0.509
	0.506	0.014	2.8	0.208	0.507
	0.509	0.010	1.9	0.609	0.5135
	0.506	0.012	2.3	0.870	0.502
	0.498	0.006	1.3	0.866	0.498
	0.495	0.012	2.5	0.631	0.4875
	0.520	0.008	1.5	0.526	0.517
	0.516	0.013	2.5	0.071	0.5125
	0.522	0.009	1.7	0.879	0.521
	0.513	0.010	1.9	0.916	0.512
Readings from the same well position of nine plates (15 wells from middle area), n = 9	0.496	0.012	2.5	0.941	0.4955
	0.499	0.011	2.2	0.874	0.502
	0.493	0.013	2.7	0.943	0.493
	0.506	0.015	2.9	0.781	0.5115
	0.498	0.019	3.7	0.774	0.4985
	0.498	0.011	2.1	0.636	0.5005
	0.488	0.015	3.1	0.319	0.485
	0.495	0.013	2.5	0.774	0.5
	0.485	0.016	3.2	0.747	0.4875
	0.487	0.009	1.9	1	0.4875
	0.486	0.016	3.3	0.220	0.4805
	0.510	0.009	1.8	0.526	0.5095
	0.508	0.009	1.7	0.952	0.509
	0.518	0.006	1.1	0.476	0.52
	0.516	0.010	2	0.126	0.521
Readings from 30 wells in each plate, n = 30	0.522	0.013	2.5	0.68	0.528
	0.506	0.017	3.4	0.779	0.528
	0.506	0.014	2.8	0.548	0.528
	0.514	0.013	2.6	0.722	0.528
	0.491	0.019	3.8	0.548	0.528
	0.501	0.017	3.5	0.28	0.528
	0.505	0.011	2.2	0.785	0.528
	0.508	0.015	3	0.628	0.528
	0.504	0.013	2.7	0.461	0.528

Culture controls were allocated to 30 wells covering marginal and middle areas of the plate and were repeated on nine plates. Assay readings were recorded (Supplementary Table S2), and standard statistics were calculated using IBM SPSS 25.

Using median values for on-plate normalization, readouts showed a skewed distribution towards negative regulation (Supplementary Table S3). In contrast, MTT readouts inclined to positive values (Supplementary Fig. S5a). Two compounds that produced abnormal DMMB readings (0.0645 ± 0.0476; − 0.0313 ± 0.0225) were considered as false positivity and thus, excluded. The other 958 compounds generated DMMB readout of 0.04612 ± 0.0418, and their CV calculation ranged from 0.05 to 21%. Indeed, nine compounds devoted high CV values (Supplementary Table S4), but the majority (99.1%) presented CV values < 10%, indicating a high reproducibility of the pre-test screening. Compounds without changing metabolic viability and exhibiting readings above or below 3 S.D. of the median (calculated from control data) were considered as enhancers or repressors, with respective hit rates of 4.48% (n = 43) and 6.04% (n = 58) (Fig. 4a) from the pre-test. These hits were evenly distributed across the MTT readouts and generated median level metabolic viability (Fig. 4b). One enhancer (E1) and one repressor (R1) were average ranked at 40–50% of their hits pool in DMMB assay but caused the least changes in MTT assay. These two hits were selected and further tested in primary chondrocytes cultured in a 3D alginate encapsulation system for GAGs production analysis^28^. Chondroitin 6-sulfate (C-6S) is reported as the major GAG produced in chondrocytes^29^. We found that C-6S could be markedly increased or reduced by the corresponding compound (Fig. 4c). Both E1 and R1 showed no cytotoxicity in alginate-cultured chondrocyte (Supplementary Fig. S5b). These results therefore support that chemical screening coupled to the DMMB assay can identify both positive and negative regulators of proteoglycans.

## Discussion

Increased and irreversible matrix breakdown in cartilaginous components is associated with the onset and progression of OA and disc degeneration^12–14^. A variety of small molecules have been discovered to promote cartilage regeneration. For example, kartogenin^21,30^ and its derivative analogue, KA34, could enhance chondrogenesis and matrix production via nuclear recruitment of the chondrogenic transcription factor CBFb. Its inhibition of matrix degradation and OA progression through induction of IL-10 was also reported^31^. SM04690, a Wnt pathway inhibitor, was reported to reduce proteoglycan degradation in differentiated chondrocytes^32^, and a phase 2B trial of SM04690 was conducted to elucidate its efficacy and safety^33^. Shi et al. demonstrated a modulatory effect of BNTA on extracellular matrices in chondrocytes via SOD3 induction^22^. These results demonstrated the value of HTS in identifying effective agents to modify musculoskeletal disorders. In this study, we investigated a miniaturized DMMB assay and optimized its adaptation for HTS library screening, aiming to identify modifiers of proteoglycan metabolism. Absorbances at 535 nm and 595 nm are two representative wavelengths for measuring GAGs in the solution sampled from the culture medium or in the digested cells^34,35^ and correspond to the formation of DMMB-GAG complexes and the consumption of the DMMB reagent in the system. Although via using chondroitin sulfate as substance, we found assay at 595 nm may give resembling absorbance window as that at 535 nm (Fig. 1a), it should be carefully considered that in the real reaction, contaminations from the cell lysate, such as DNA, may also bind with DMMB and interfere the readings at 595 nm^23^. It is intriguing that at 620 nm, a larger absorbance window was observed. How well this wavelength serves the DMMB assay awaits to be further addressed. A more concentrated DMMB reagent was used to generate a wider linear reading window (0–664 μg/mL, x-coordinate in Fig. 2b) and a larger absorbance scale (0.39–0.66, y-coordinate in Fig. 2b). Overall, several modifications to the conventional assay method that are critical to effective HTS were implemented: (1) Medium refreshment and cell digestion prior to the DMMB reaction were avoided for an efficient high-throughput workflow. (2) A_450nm_ was measured as a reference for A_535nm_ to minimize interferences arising from the plate and buffers. (3) Measurements were acquired 5 min instead of immediately after DMMB addition to obtain more steady readouts, therefore minimizing variation during large batch processing.

We selected porcine costal chondrocytes over cell lines such as ATDC5^22^ or MSC-derived chondrocytes^32^ for assay development. We reasoned that primary chondrocytes provide a more native phenotype, in particular matrix production, for functional screening. A relatively high seeding density of 3 × 10^4^ cells/cm^2^ was adopted to maintain the cells in a differentiated state, as indicated by the cobblestone morphology typically observed in mature chondrocytes. Furthermore, assay kinetics were determined to identify midpoints of the linear range for maximizing the detection of both up- and downregulation of GAG production. The MTT assay was optimized to evaluate global metabolism and was applicable as a reference for assessing regulatory specificity in proteoglycan metabolism. However, with longer incubation times, such readings gradually increase, and twenty-four hours seems adequate to maximize the readings.

TGF-β1 is an anabolic regulator of proteoglycans and an inhibitor of their degradation, whereas IL1-α is a catabolic effector^25,26^. The results from TGF-β1- and IL1-α-treated cells substantiate the capacity of the system to detect up- and downregulation of GAGs. A Z′ score over 0.5 is considered “excellent” for signal-to-noise separation^27^, and our assay was shown to be unbiased for identifying both positive and negative signals and indicative of HTS compatibility. Based on analysis of the medium controls, our data demonstrated no significant plate-to-plate variation, suggesting the feasibility of cross-plate comparison. In this study, the median from each plate was exploited for normalization to further increase the assay robustness.

A proof-of-concept chemical screening was conducted using the miniaturized DMMB assay, and both positive and negative regulatory candidates were identified from the library. The majority of the 960 compounds (79.69%) showed readouts within 3 × S.D., which were considered insignificant^36^. Notably, the screening results showed a skewed distribution, and a larger panel of compounds with < 3 × S.D was identified (15.10% vs 5.21%). This is consistent with the known tendency of target inhibition of small molecules. Interestingly, the screening using MTT assay also demonstrated a skewed distribution, but towards positive readouts, implying the intensive bioactivity of the compounds pool (Supplementary Table S3). It should be noted that the library is small, and thus, the screening process is primitive and not considered high throughput. However, the chemical screening was conducted with the automated platform, and as a result could be readily adapted to large-scale study (e.g. 50 k compound library) based on our previous experience^37^. The hit rate is considered relatively high (20.31%), presumably due to the broad cut-off and false positivity. MTT assay allows evaluating not only cellular toxicity but also  global metabolic activity. In this study, evaluation of metabolic viability by MTT assay was implemented to narrow down the hits (4.48% vs 6.04%, enhancers vs repressors) which can specifically regulate proteoglycans production without altering metabolic viability. While top-ranked compounds are usually prioritized based on the primary readouts in HTS, we selected E1 and R1 to better assess the assay reliability in view of their moderate regulatory effect on proteoglycans production among the hit. Besides, they both caused minimal MTT changes, implying a specific instead of global regulation. Sequential screenings are typically required to narrow down the hits, where various concentrations of compounds can be included to evaluate dose-dependency. Moreover, cheminformatic clustering is widely employed to assess the molecular fingerprint similarity of a large panel of compounds and could jointly facilitate the hit selection process. The reproducibility in triplicates is supported by CV values < 10% from 99.1% compounds. False positives, such as due to the compound's intrinsic colour should be examined and removed in subsequent screening. In this scenario, readings from the compound   might not follow a linear relationship with dosage  and further sample processing for compound-free validation may be required. Taking our work as an example, GAGs can be purified from culture after treatment and quantified by FACE. Moreover, notwithstanding the significant changes detected in chondroitin sulfate expression, it is not clear whether other types of GAGs was also modulated, such as keratan sulfate, the other major GAG component in aggrecan and SLRP that is highly expressed in chondrocytes. There also might be compounds that alter the GAG composition without changing the total GAG content and therefore not detected by the assay.

Identifying both stimulators and inhibitors of proteoglycan metabolism can have relevant implications. Proteoglycans play critical roles in signalling activation^6,8,10^, tissue development and morphogenesis^3,18,20^ and participate in pathological processes other than OA, such as tissue fibrosis^38^ and outgrowth of dorsal root ganglion (DRG) axons in discogenic pain^4^. While GAG repressors are likely not relevant to cartilage regeneration, their identification might potentially be beneficial to axonal regeneration for peripheral nerve or spinal cord repair^4,39^.

In conclusion, we established an enhanced, miniaturized DMMB assay and illustrated its effective integration with HTS platform and chemical screening to identify regulators of GAG expression using chondrocytes as a model. Proteoglycan production is rudimentary to chondrocyte function. GAG-based primary screening may therefore provide a simple way to prioritize leads in a large library for further investigation. Our study serves as a proof-of-principle for quantitative GAG-based HTS screening. Its application may facilitate the identification of new agents for treating OA and intervertebral disc degeneration and possibly other disorders related to proteoglycan misregulation. Furthermore, considering the importance of GAGs in the polymerization and activation of signalling molecules, such as STING^10^, screening GAG regulators may tap into new resources for manipulating these pathways.

## Methods

### Chondrocyte extraction and expansion

Fresh costal cartilage tissues from large white pigs were provided by the Laboratory Animal Unit, The University of Hong Kong. Primary chondrocytes were then isolated through sequential enzyme digestion^24^, expanded at high density in high-glucose DMEM supplemented with 10% foetal calf serum (FCS, Biosera, FR), 4.5 mg/mL l-glutamine, 1% penicillin/streptomycin, 0.4% fungizone and 1 µg/mL gentamycin, with media refreshed every 2 days at 37 °C at 5% CO_2_ and cultivated until passage 2 (P2) for HTS.

### High-throughput assay development

The optimization of miniaturized DMMB (1,9-dimethyl-methylene blue) and MTT (thiazolyl blue tetrazolium bromide) assays was carried out in 96-well plates and later scaled to 384-well plates for HTS applications. The DMMB reaction was carried out by adding an equal volume of DMMB assay buffer directly to the wells without removing the culture medium, and the absorbance was recorded using DTX 800 Series Multimode Detectors (Beckman Coulter, Inc.). Reference wavelength determination was implemented by discontinuous spectroscopy at 360, 405, 450, 485, 535, 595, and 620 nm with increasing concentrations of chondroitin 4-sulfate (2–1128 μg/mL). For maximal assay linearity, gradient DMMB reagents of 1×, 3×, and 5× were prepared by dissolving 16, 48 and 80 mg DMMB in 1 L of deionized water containing 10.64 g of glycine and 9.35 g of NaCl (pH 3.0). The reaction kinetics were evaluated to allow DMMB assay measurement up to 16 min of incubation. Next, chondrocytes (P2) were seeded at densities of 6 × 10^4^, 5 × 10^4^, 4 × 10^4^, 3 × 10^4^, and 2 × 10^4^ cells/cm^2^ in 50 μL of complete medium without phenol red for up to 4 days, and cell morphology was recorded daily under microscope. Absorbance was measured after DMMB dye addition without change of medium .

For the MTT assay, 20 μL of MTT (0.63 μg/mL) was added to the 50-μL culture system, of which chondrocytes were initially seeded at 3 × 10^4^ and 2 × 10^4^ cells/cm^2^, and the mixture was incubated at 37 °C in dark for 8 to 32 h. The reaction was terminated by adding 30 μL of 10% SDS and monitoring the absorbance at a wavelength of 570 nm with reference to 640 nm.

TGF-β1- or IL1-α-treated samples were used as positive or negative controls for evaluating assay performance ^25,26^. With the aforementioned setup, chondrocytes were treated with either TGF-β or IL1-α  at 10 ng/mL for 3 days.  DMMB assay was performed using the optimized conditions.

### Plate uniformity assay

Plate uniformity was evaluated as previously described to investigate the signal variability across different plates^36^. A library containing 960 small molecules (ChemBridge Co., US) with molecular weights ranging between 160 and 6600 Da was arrayed on three plates (320 compounds/plate) in triplicate to assess the assay for screening in high-throughput mode. A multidrop well liquid dispenser (MTX Lab System, Inc.) was used for the addition of all reagents to the assay plates. Twenty-five microlitres of complete medium without phenol red was added to 384-well polystyrene microtiter plates, followed by pin-arraying the compounds at 20 μg/mL,  pre-dissolved in DMSO (Hybri-Mix, Sigma, US), into the plates via an automation platform (Biomek^®^ 3000, Beckman Coulter). Porcine chondrocytes were dispensed at 3 × 10^4^ cells/cm^2^ in 25 μL of complete DMEM. In each plate, the first and last two columns were reserved for nontreated controls (0.1% DMSO). The plates were incubated at 37 °C in 5% CO_2_ for 3 days, followed by assessment of cell viability and GAG production using MTT and DMMB assays.

### Fluorophore-assisted carbohydrate electrophoresis (FACE)

The two hit compounds obtained from HTS were validated in alginate-cultured chondrocytes^24^. In brief, chondrocytes were encapsulated in 1.2% alginate hydrogel (Sigma, US) at a seeding density of 2 × 10^6^ cells/mL (approximately 4 × 10^4^ cells in each bead) and maintained in complete DMEM supplemented with 50 μg/mL ascorbic acid for 7 days. The culture was treated  with the two compounds for another three days. Alginate cultured cells were further incubated with MTT for 3 h. MTT formazan were then released by papain digestion, precipitated by high-speed centrifuge and further dissolved by DMSO. Absorbance at 490 nm was measured. GAGs in the supernatant were precipitated by DMMB reagent (1×) and were further released in dissociation buffer (38.1 g of guanidine hydrochloride and 0.68 g of sodium acetate trihydrate in 100 mL of dH_2_O containing 10% propan-1-ol). Next, GAGs were purified by ethanol precipitation and digested with chondroitinase ABC (100 mU/mL, from Proteus vulgaris, Seikagaku, JP). Chondroitin sulfate (CS) disaccharides were tagged with fluorescent 2-aminoacridone (dAMAC), fractionated in 25% polyacrylamide gels and quantified using the gel densitometry function of ImageJ (Version 1.46r, National Institutes of Health, US).

### Data analysis

Data were analysed using IBM SPSS 25 software and shown as means ± standard deviations (S.D.). A trendline was generated for R^2^ calculation during assay development. The means and S.D. of positive (TGF-β) and negative (IL1-α) controls  were used for assay performance analysis  of Z′ with respect to on-plate controls, of which > 0.5 was indicative of the suitability of the assays for HTS. In screening, the readout of each compound was subtracted from the median value in each plate for cross-plate comparison. Compounds that showed normalized values above or below 3 S.D. of the median of the corresponding plate were considered enhancers and repressors, respectively.

## Supplementary Information

Supplementary Information.
