# Supplementary material for: Integration of a miniaturized DMMB assay with high-throughput screening for identifying regulators of proteoglycan metabolism
Source: Sci Rep. 2022 Jan 20;12:1083. doi: 10.1038/s41598-022-04805-y (PMC8776954; doi:10.1038/s41598-022-04805-y)
Supplement: Supplementary file 1 — Supplementary Information. [file 41598_2022_4805_MOESM1_ESM.docx]

Title:

Integration of miniaturized DMMB assay with high-throughput screening for identifying regulators of proteoglycan metabolism

**Author list:**

**Yi Sun**^†^**,** Department of Orthopaedics and Traumatology, The University of Hong Kong, China

Yuen-kee Tsui^†^, Department of Orthopaedics and Traumatology, The University of Hong Kong, China

Mengqi Yu, Department of Diagnostic Radiology, National University of Singapore, Singapore

Minmin Lyu, Core laboratory, The University of Hong Kong Shenzhen Hospital, China

Kenneth Cheung, Department of Orthopaedics and Traumatology, The University of Hong Kong, China

Richard Kao*, Department of Microbiology, The University of Hong Kong, China

Victor Leung*, Department of Orthopaedics and Traumatology, The University of Hong Kong, China

**Supplementary Table S1.** Linearization characterization of different DMMB reagent concentration in the assay.

| **Reagent conc**. | **Reading window** | **Trendline slope** | **Trendline R^2^** |
| --- | --- | --- | --- |
| 5x | 0-64 | 0.0043 | 0.993 |
| 4x | 0-64 | 0.0036 | 0.9327 |
| 3x | 0-32 | 0.0051 | 0.9962 |
| 2x | 0-32 | 0.0047 | 0.9988 |
| 1x | 0-16 | 0.0046 | 0.9961 |

**Supplementary Table S2. Assay readings of culture controls from the chemical screening.**

|  | **DMMB reading** | | | | | | | | |
| --- | --- | --- | --- | --- | --- | --- | --- | --- | --- |
|  | Plate-1 | Plate-2 | Plate-3 | Plate-4 | Plate-5 | Plate-6 | Plate-7 | Plate-8 | Plate-9 |
| Edge  wells | 0.515 | 0.510 | 0.511 | 0.489 | 0.471 | 0.513 | 0.506 | 0.516 | 0.526 |
|  | 0.542 | 0.512 | 0.513 | 0.521 | 0.516 | 0.518 | 0.517 | 0.531 | 0.522 |
|  | 0.534 | 0.520 | 0.507 | 0.526 | 0.493 | 0.540 | 0.516 | 0.521 | 0.511 |
|  | 0.555 | 0.551 | 0.525 | 0.530 | 0.525 | 0.530 | 0.534 | 0.542 | 0.523 |
|  | 0.542 | 0.540 | 0.516 | 0.537 | 0.494 | 0.530 | 0.521 | 0.511 | 0.503 |
|  | 0.521 | 0.515 | 0.514 | 0.519 | 0.493 | 0.508 | 0.508 | 0.520 | 0.525 |
|  | 0.533 | 0.508 | 0.509 | 0.512 | 0.481 | 0.509 | 0.497 | 0.508 | 0.498 |
|  | 0.528 | 0.506 | 0.519 | 0.512 | 0.505 | 0.506 | 0.499 | 0.502 | 0.500 |
|  | 0.522 | 0.512 | 0.508 | 0.512 | 0.482 | 0.503 | 0.501 | 0.515 | 0.496 |
|  | 0.501 |  | 0.507 | 0.495 |  | 0.501 | 0.489 |  | 0.494 |
|  | 0.506 | 0.498 | 0.507 | 0.498 | 0.468 | 0.494 | 0.499 | 0.503 | 0.485 |
|  | 0.529 | 0.523 | 0.528 | 0.524 | 0.505 | 0.513 | 0.517 | 0.526 | 0.518 |
|  | 0.528 | 0.525 | 0.527 | 0.529 | 0.496 | 0.509 | 0.505 | 0.523 | 0.502 |
|  | 0.524 | 0.517 | 0.531 | 0.535 | 0.522 | 0.514 | 0.507 | 0.530 | 0.517 |
|  | 0.527 | 0.497 | 0.514 | 0.522 | 0.516 | 0.500 | 0.512 | 0.517 | 0.510 |
| Middle  wells | 0.515 | 0.498 | 0.491 | 0.509 | 0.476 | 0.484 | 0.504 | 0.491 | 0.496 |
|  | 0.519 | 0.490 | 0.497 | 0.508 | 0.485 | 0.487 | 0.498 | 0.503 | 0.508 |
|  | 0.514 | 0.495 | 0.499 | 0.503 | 0.472 | 0.474 | 0.495 | 0.493 | 0.490 |
|  | 0.519 | 0.487 | 0.494 | 0.536 | 0.503 | 0.506 | 0.504 | 0.495 | 0.511 |
|  | 0.530 | 0.509 | 0.492 | 0.509 | 0.467 | 0.479 | 0.509 | 0.497 | 0.493 |
|  | 0.518 | 0.483 | 0.491 | 0.503 | 0.492 | 0.493 | 0.497 | 0.497 | 0.510 |
|  | 0.512 | 0.493 | 0.483 | 0.502 | 0.458 | 0.479 | 0.491 | 0.493 | 0.481 |
|  | 0.520 | 0.480 | 0.489 | 0.508 | 0.494 | 0.485 | 0.494 | 0.486 | 0.500 |
|  | 0.502 | 0.478 | 0.492 | 0.514 | 0.461 | 0.475 | 0.484 | 0.480 | 0.481 |
|  | 0.503 | 0.472 | 0.486 | 0.496 | 0.484 | 0.479 | 0.490 | 0.485 | 0.492 |
|  | 0.504 | 0.503 | 0.477 | 0.488 | 0.457 | 0.473 | 0.502 | 0.493 | 0.478 |
|  | 0.522 | 0.503 | 0.497 | 0.518 | 0.501 | 0.504 | 0.519 | 0.514 | 0.509 |
|  | 0.512 | 0.513 | 0.508 | 0.522 | 0.497 | 0.503 | 0.506 | 0.516 | 0.496 |
|  | 0.531 | 0.518 | 0.517 | 0.520 | 0.514 | 0.509 | 0.516 | 0.519 | 0.518 |
|  | 0.539 | 0.510 | 0.518 | 0.508 | 0.503 | 0.517 | 0.519 | 0.517 | 0.513 |

**Supplementary Table S3. Statistics of the chemical screening.** 960 compounds were randomly allocated to 3 plates and assayed in triplicates. Skewness and Kurtosis were evaluated using IBM SPSS 25. A skewed distribution towards negative (DMMB) or positive readouts (MTT) were observed.

|  | | **Plate-1** | | | **Plate-2** | | | **Plate-3** | | |
| --- | --- | --- | --- | --- | --- | --- | --- | --- | --- | --- |
| **DMMB assay** | **Skewness** | -5.056 | -3.254 | -4.061 | -4.047 | -3.887 | -3.407 | -1.321 | -1.332 | -1.503 |
|  | **Kurtosis** | 41.352 | 18.715 | 27.766 | 31.49 | 30.997 | 22.491 | 1.969 | 2.048 | 2.529 |
| **MTT**  **assay** | **Skewness** | 2.643 | 3.464 | 3.296 | 1.107 | 1.057 | 1.215 | 1.089 | 1.016 | 1.016 |
|  | **Kurtosis** | 12.618 | 17.162 | 15.143 | 3.130 | 2.449 | 3.201 | 3.137 | 3.136 | 2.914 |

**Supplementary Table S4. Summarization of 9 compounds with CV > 10% in pre-test screening.** These compounds were arrayed in triplicates in both DMMB and MTT assays. Their normalized readings were summarized. None of them were regarded as hits.

| Compound ID | DMMB readouts | | | MTT readouts | | |
| --- | --- | --- | --- | --- | --- | --- |
| S02331 | 0.3034 | 0.2055 | 0.2232 | 0.3586 | 0.3370 | 0.3506 |
| S02369 | 0.5362 | 0.4192 | 0.4773 | 0.3653 | 0.3705 | 0.3823 |
| S02159 | 0.4838 | 0.5519 | 0.4514 | 0.3598 | 0.3158 | 0.3270 |
| S07321 | 0.5546 | 0.4629 | 0.4716 | 0.3420 | 0.3213 | 0.3622 |
| S07247 | 0.3769 | 0.4010 | 0.3171 | 0.2129 | 0.2584 | 0.2607 |
| S07004 | 0.3706 | 0.3852 | 0.3099 | 0.8873 | 0.8617 | 0.8828 |
| S12293 | 0.2657 | 0.3439 | 0.3334 | 0.3656 | 0.3515 | 0.4478 |
| S12083 | 0.3669 | 0.3896 | 0.4667 | 0.4808 | 0.5145 | 0.5037 |
| S12343 | 0.4553 | 0.4888 | 0.5744 | 0.1873 | 0.1840 | 0.2096 |

**Supplementary Figure S1. Kinetics of MTT assay with two seeding densities.** Values are presented as the mean ± SEM of triplicates.

**
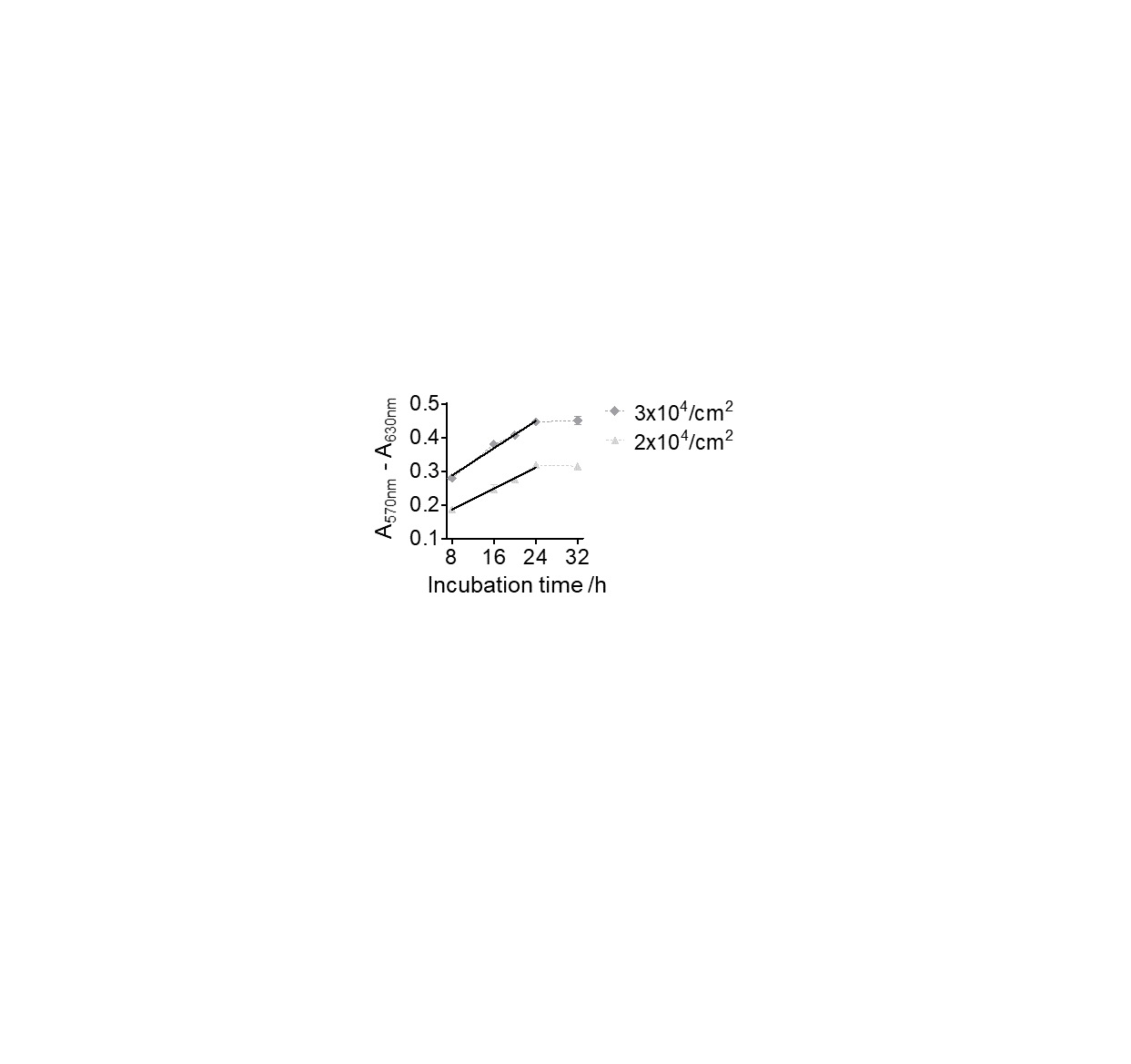
**

**Supplementary Figure S2. Supplementary Figure S2. Schematic overview of the high-throughput DMMB assay.** Chondrocytes were seeded and incubated with small molecules-containing medium for 72 hours. Culture mixtures were then incubated with equal volume of concentrated DMMB reagent for 10 min prior to absorbance reading. Image was generated in Office 365 Power Point.

**
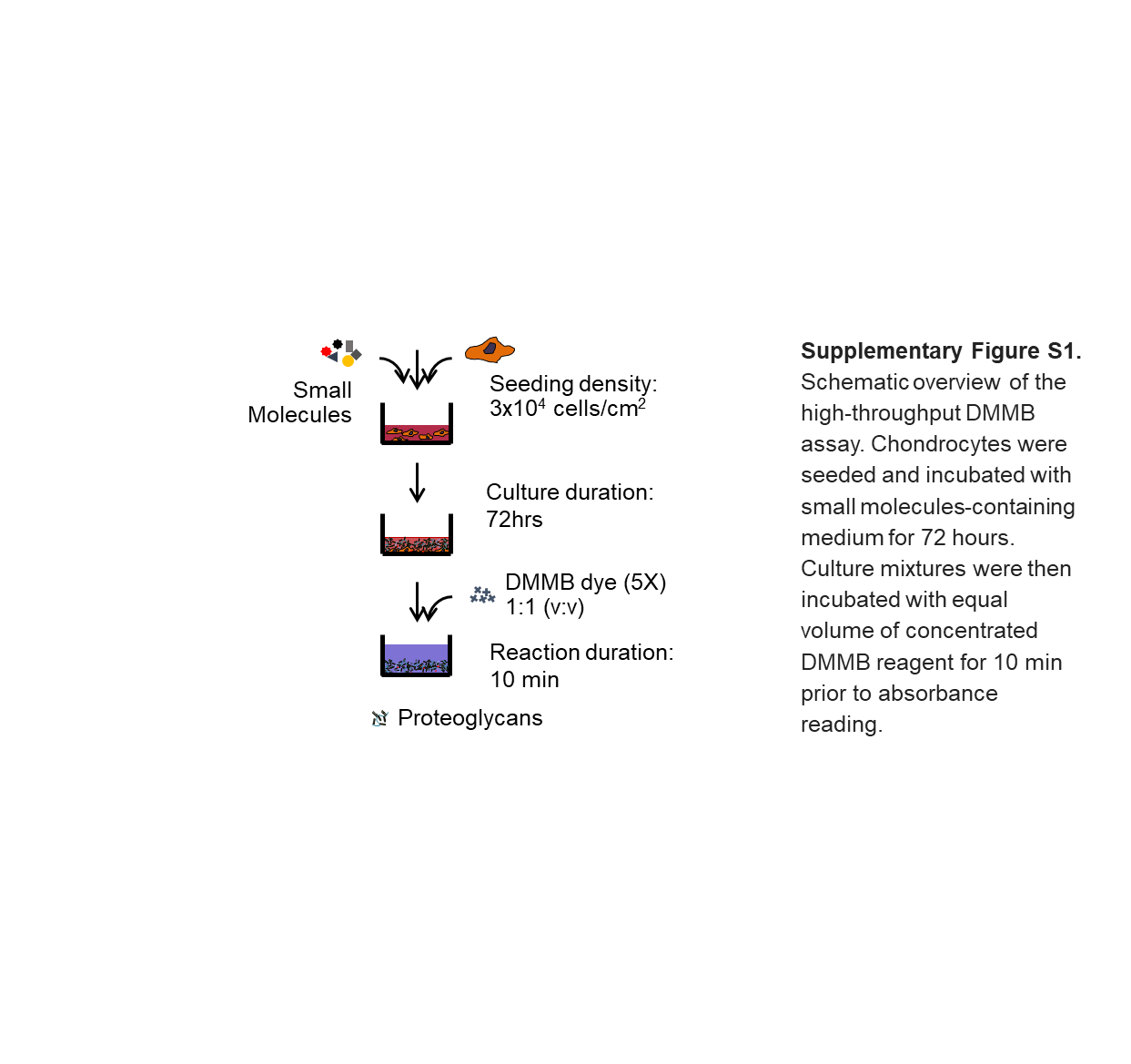
**

**Supplementary Figure S3. Statistics of culture controls in HTS assay.** Medium control was arrayed in wells from edge (0-15) and middle (16-30) areas of in total nine independent plates. (a) Scatter plots of DMMB assay readout from each well. (b) Z scores from each plate. Colors represent plates.


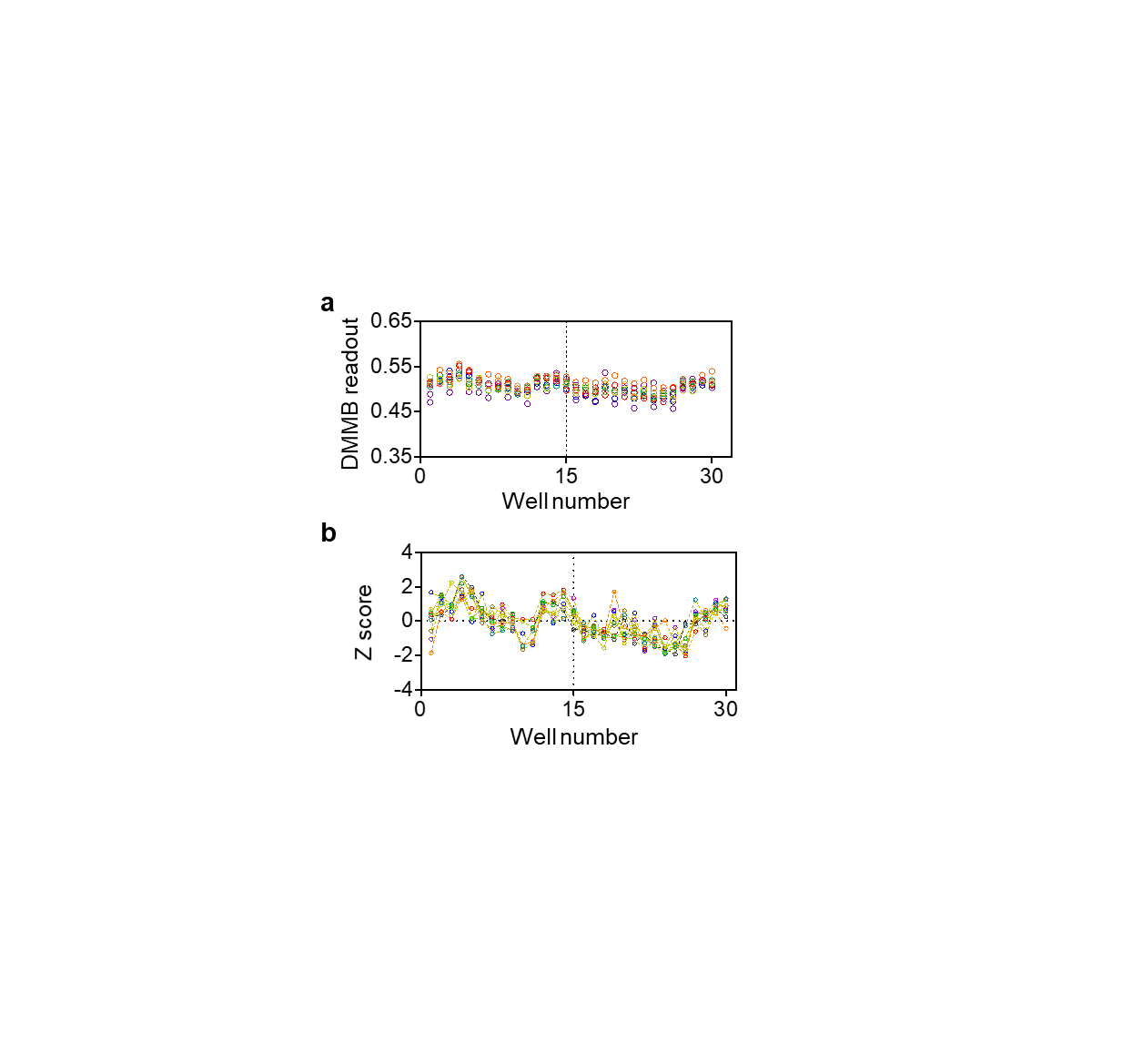


**Supplementary Figure S4. Original FACE images for Figure 4b.** Cropped areas were highlighted. Disaccharides standards were marked in line S1, S2 and S3.


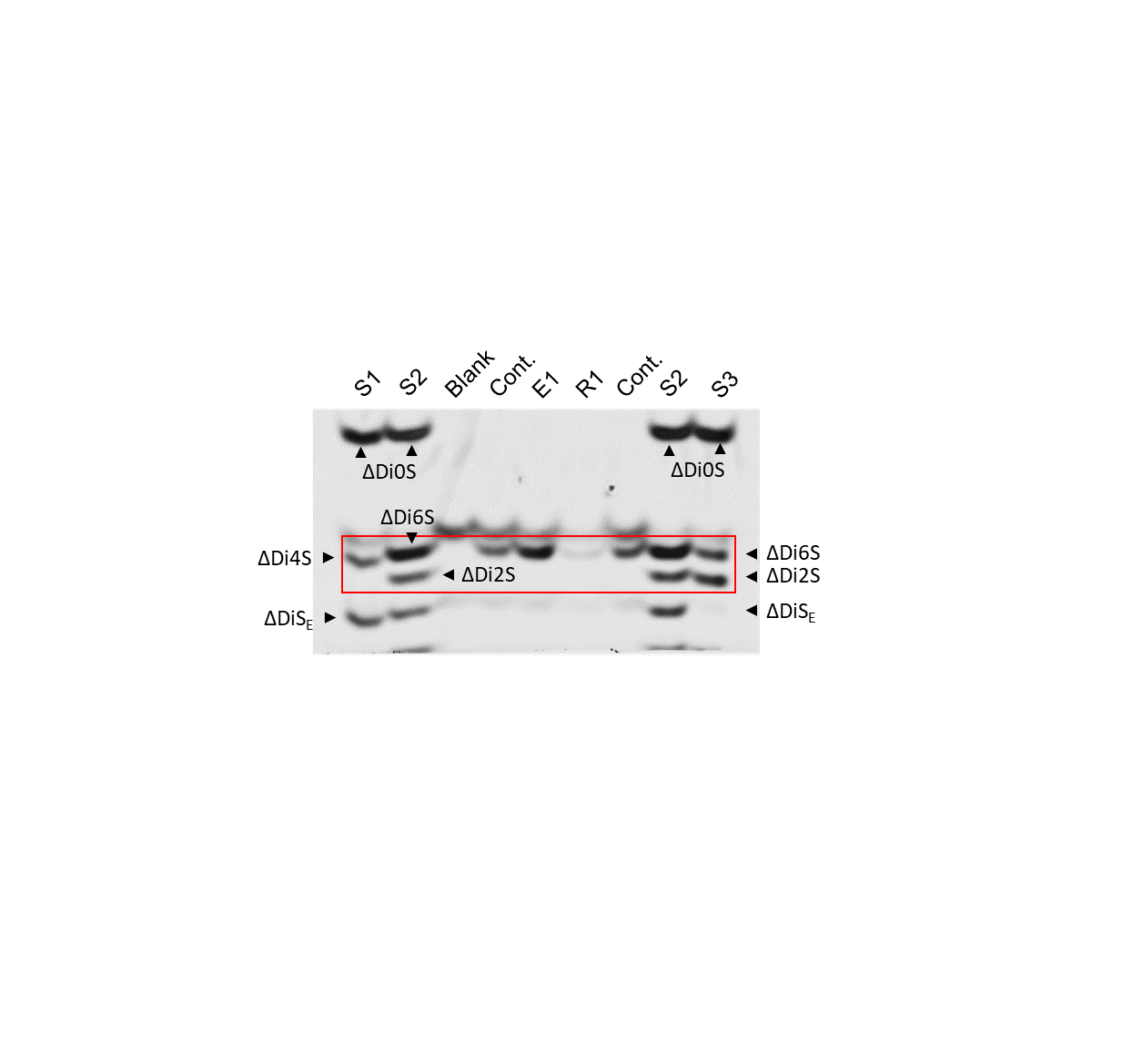


**Supplementary Figure S5. Metabolic viability of 960 compounds via the HTS MTT assay. (a)** Scatter plot of MTT assay readouts from 960 compounds. The median of each plate was used for subtraction normalization. Each batch contained 320 compounds and was performed in triplicate. Enhancer (E1) and repressor (R1) from HTS DMMB assay were marked. **(b)** Cytotoxicity assessment of E1 and R1 in alginate-cultured primary chondrocytes by MTT assay. Representative MTT colorimetric image was inserted.

*
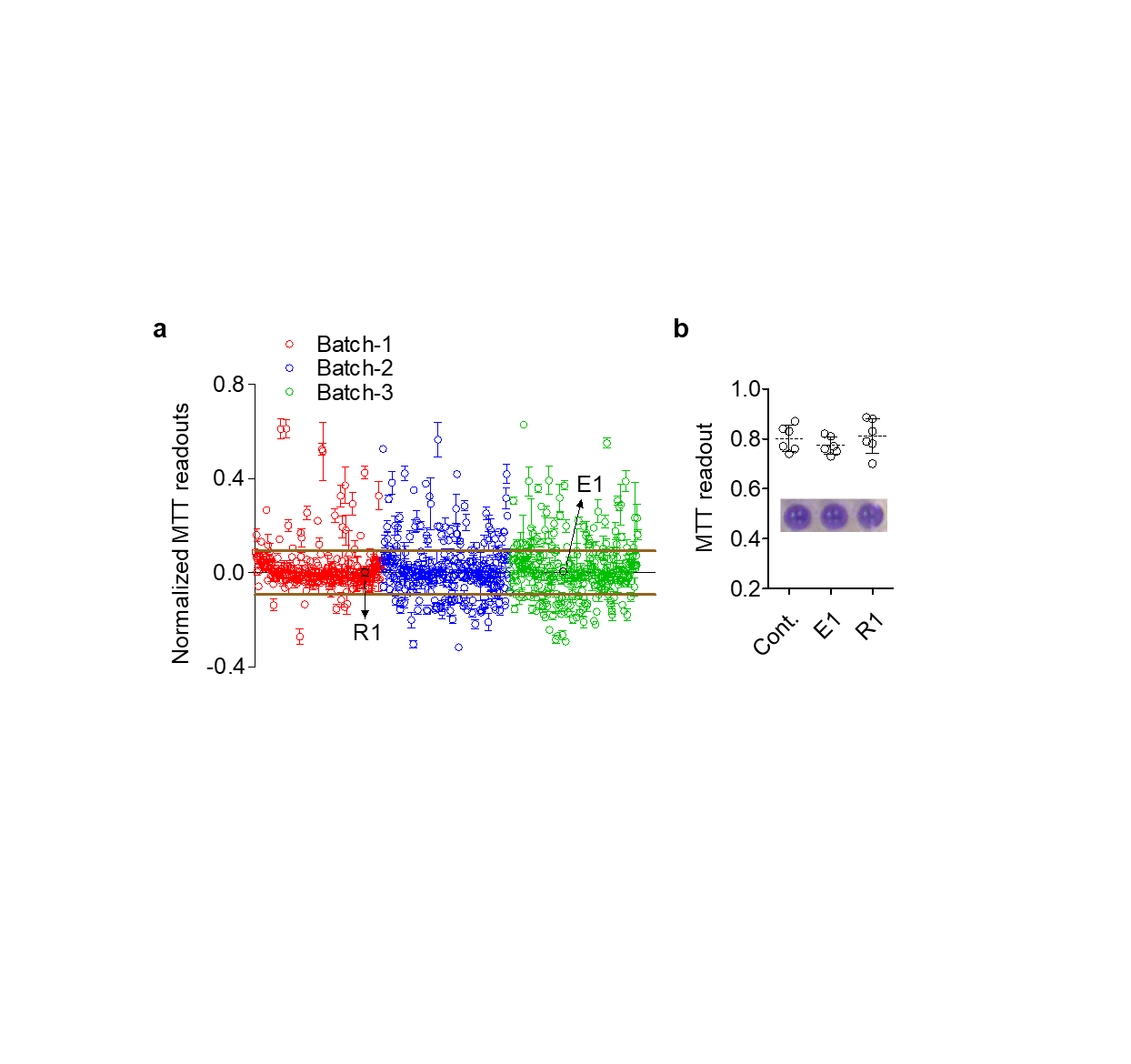
*
